# Brown Tumor With Spine Involvement at Multiple Levels in a Hemodialysis Patient

**DOI:** 10.7759/cureus.17000

**Published:** 2021-08-08

**Authors:** Victoria Ghernautan, Zarwa Idrees, Mahmoud Nassar, Camelia Ciobanu, Adesh Ramdass

**Affiliations:** 1 Internal Medicine, Icahn School of Medicine at Mount Sinai/NYC Health+Hospitals Queens, New York, USA; 2 Medicine, St. Barnabas Hospital, The Bronx, USA; 3 Medicine, Icahn School of Medicine at Mount Sinai/NYC Health+Hospitals Queens, New York, USA

**Keywords:** brown tumor, secondary hyperparathyroidism, bone lytic lesions, chronic kidney disease, parathyroid hormone, osteitis fibrosa cystica, vertebral brown tumor

## Abstract

Brown tumor of the bone or osteitis fibrosa cystica is a rare manifestation of hyperparathyroidism, most seen nowadays in association with secondary and tertiary hyperparathyroidism. Chronic kidney disease (CKD) and end-stage renal disease (ESRD) are the major culprits of secondary hyperparathyroidism (sHPTH). CKD is known to cause phosphate retention and a decrease in 1,25-dihydroxyvitamin D and ionized calcium levels, which in turn trigger the PTH secretion. Brown tumor can affect the jawbones, femur, sternum, ribs, and rarely the spine. We present the case of a 60-year-old male with ESRD on hemodialysis who was found to have lytic bone lesions in the thoracic and lumbar spine. Initially, malignancy was suspected. Blood work revealed markedly elevated PTH at 3,563 pg/mL, hypocalcemia, and hyperphosphatemia. Biopsy of the L5-S1 lesion was consistent with reactive changes due to sHPTH. Once a diagnosis of the brown tumor was confirmed, the patient was started on cinacalcet and was referred for parathyroidectomy.

## Introduction

Metabolic bone disease in chronic kidney disease (CKD) is a result of several abnormalities in calcium, phosphorus, and vitamin D metabolism. Decreased ionized calcium and hyperphosphatemia stimulate parathyroid hormone (PTH) secretion, promoting the development of secondary hyperparathyroidism (sHPTH) [1]. Persistently elevated PTH increases osteoclastic activity, bone resorption, and breakdown resulting in osteitis fibrosa cystica aka brown tumor [2]. Brown tumor is a rare complication of CKD, especially in the Western world, due to the early detection and treatment of hyperparathyroidism. It can occur in any bone; however, spine involvement is exceedingly rare [3-5]. Here, we describe a rare case of brown tumor involving the thoracic and lumbosacral spine in a patient on hemodialysis for end-stage renal disease (ESRD).

## Case presentation

A 60-year-old African American male with a past medical history of essential hypertension, hyperlipidemia, coronary artery disease, bilateral knee osteoarthritis, ESRD on hemodialysis three times a week and uncontrolled sHPTH presented to the emergency room complaining of back pain for a week after a fall. The pain was sharp, constant, radiating to the bilateral hips. The pain was worse with movement and was alleviated by acetaminophen. The patient reported compliance with the scheduled hemodialysis treatments. His home medication regimen consisted of low-dose aspirin, calcitriol 0.25 mcg daily, furosemide 40 mg twice a day, hydralazine 100 mg every eight hours, nifedipine ER 90 mg daily, and sevelamer 800 mg three times a day.

There were lytic bone lesions in the seventh rib, T9, T11, and L1 vertebrae incidentally found on a CT chest two months prior during an ER visit. The patient was referred to an oncology outpatient clinic. He did not make it to the oncology clinic as he needed medical attention for the back pain.

On examination, midline lower back tenderness was elicited, and no neurological deficits were found. The presence of the lytic lesions on previous CT prompted our team to suspect metastatic cancer or multiple myeloma. Oncology service was consulted and recommended an urgent MRI of the lumbar spine due to the concern for cord compression, and CT of the abdomen and chest with contrast to localize other potential bone lytic lesions. They also recommended a biopsy of the most accessible lytic lesion.

Blood work showed normocytic anemia, normal leukocytes, elevated blood urea nitrogen (BUN) and creatinine, mildly elevated kappa and lambda free light chains, normal serum protein electrophoresis, normal prostate-specific antigen (PSA), normal liver enzymes, and bilirubin. Additional tests showed decreased ionized calcium (1.12 mmol/L), markedly elevated PTH at 3,563 pg/mL, alkaline phosphatase 183 U/L (N, 40-129 U/L), and phosphorus 6.9 mg/dL (N, 2.5-4.5 mg/dL).

**Figure 1:**
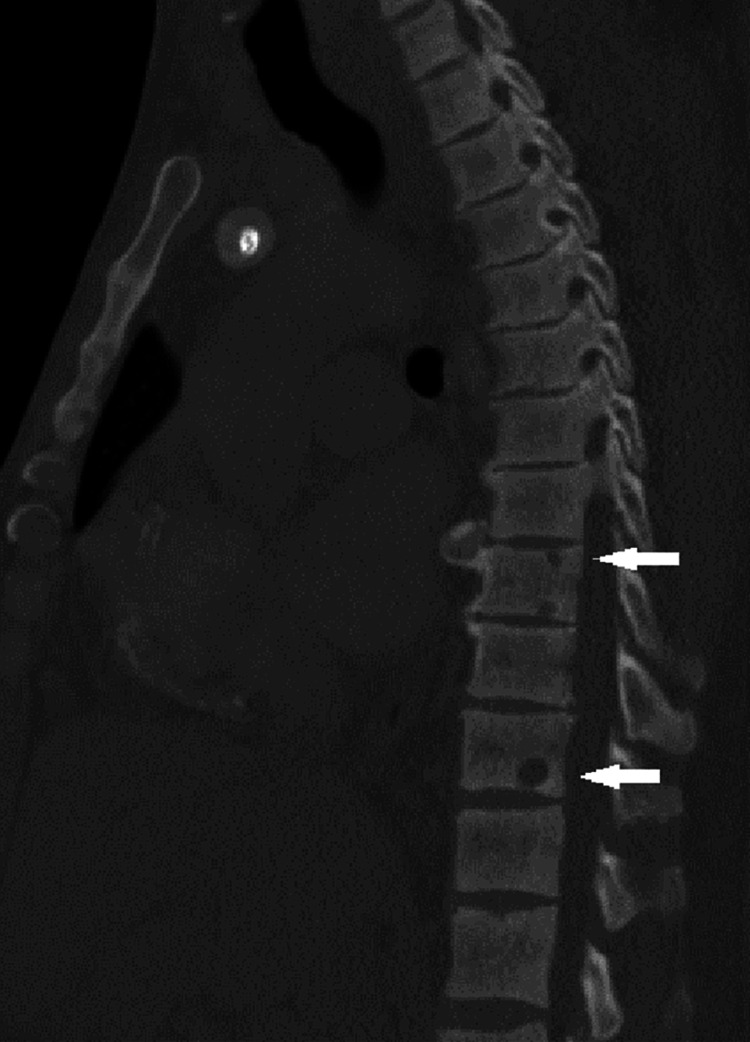
CT of the chest with contrast showing the bone lytic lesions in the thoracic vertebrae.

**Figure 2:**
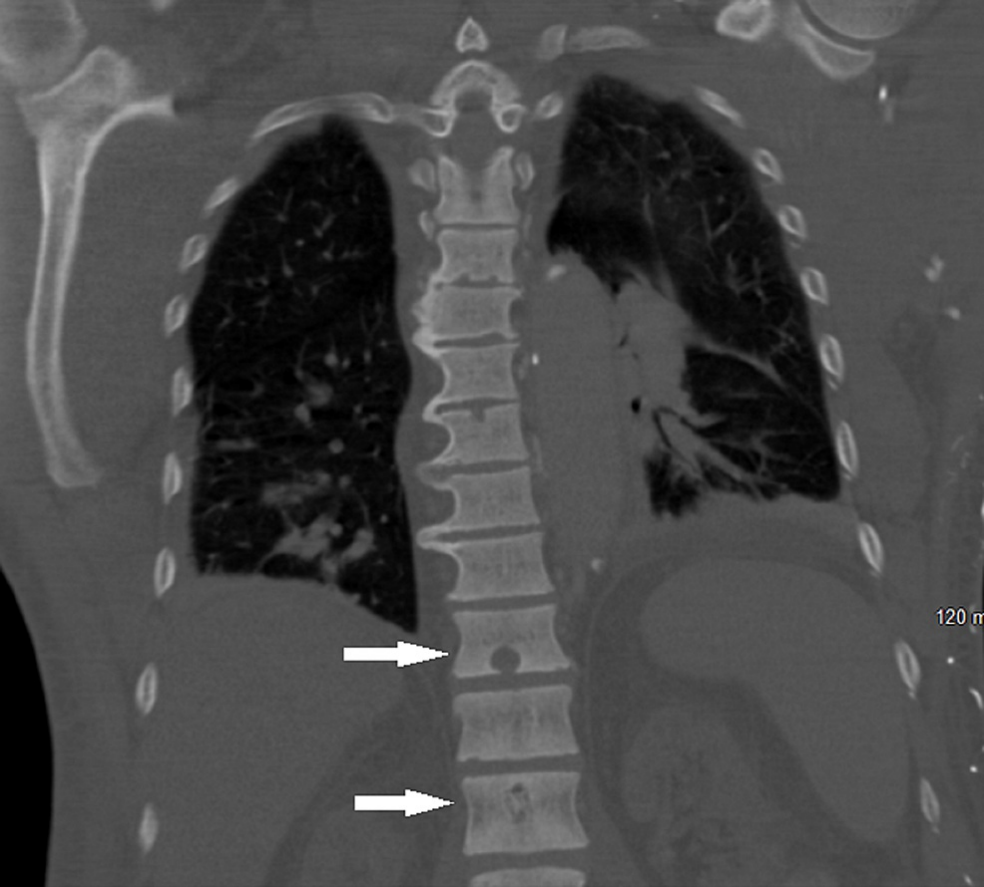
CT of the chest with contrast showing the bone lytic lesions in the thoracic vertebrae and L1 vertebra.

**Figure 3:**
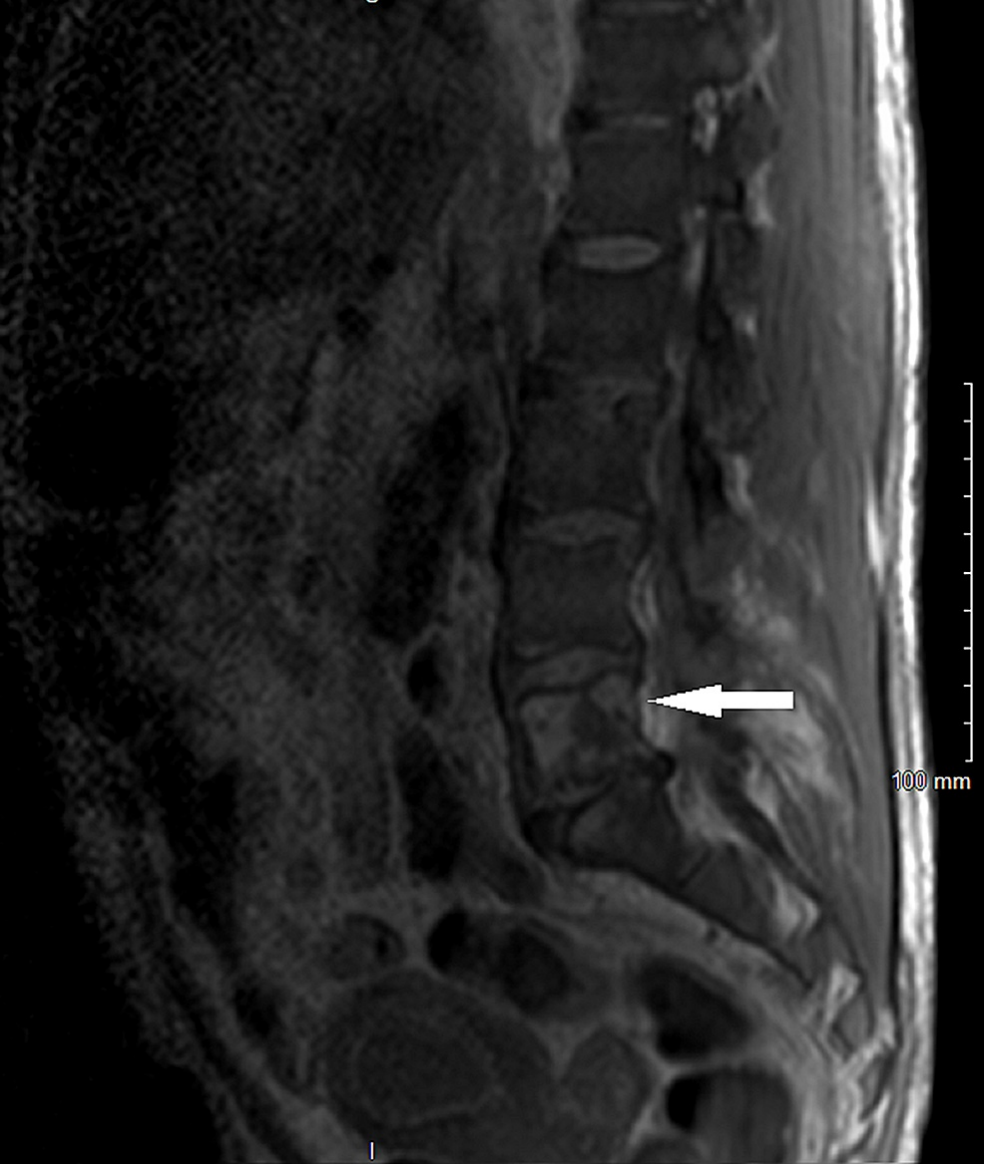
MRI of the lumbar spine without contrast showing the L5 lytic lesion.

MRI of the lumbar spine showed no evidence of cord compression. Abdominal and chest CT identified the lytic lesions in the thoracic T9, T11 (Figures 1, 2), lumbar L1, L5 (Figure 3), and sacral S1 spine, and the seventh rib, which were described as suspected brown tumors but no other findings to support primary cancer or organ metastases. Total body bone scintigraphy was strongly suggestive of metabolic bone disease.

Ultrasound-guided fine-needle aspiration of the L5-S1 lytic lesion showed chronic inflammation, fibrosis, and spindle cells consistent with reactive changes. The clinical picture along with the radiographic findings and cytology was supportive of brown tumor diagnosis and less likely of malignancy such as multiple myeloma or giant-cell tumor of the bone.

Nephrology service was also involved in patient’s care and they recommended to continue the phosphate binder and switch to cinacalcet from calcitriol. He was also referred for parathyroidectomy.

The patient followed up in the primary care clinic, he was still complaining of back pain. He has a scheduled appointment with ENT for evaluation for parathyroidectomy.

## Discussion

ESRD is one of the most common causes of sHPTH and can lead to the so-called “renal osteodystrophy.” It includes several manifestations such as osteitis fibrosa cystica, osteomalacia, osteosclerosis [4,6]. Osteitis fibrosa cystica can present as lytic or sclerotic bone lesions secondary to severe bone resorption due to persistently high levels of PTH [4]. They are called brown tumors and occur more often in the jaw, ribs, sternum, and long bones. Several cases of spine involvement were described; however, they are rare [3-5]. Moreover, multilevel vertebral brown tumors are extremely rare [7]. In certain cases, they can be recurrent [8]. The first case of a brown tumor of the spine secondary to primary hyperparathyroidism (pHPTH) was published in 1968 [6].

Brown tumor is a benign and reactive lesion secondary to disrupted bone remodeling, which can occur in both pHPTH and sHPTH. It develops in patients with extremely elevated PTH levels for a prolonged period of time [2,9]. The incidence of brown tumors in ESRD patients on HD is up to 13% [4,9,10], as opposed to 1.5% in pHPTH [9].

Brown tumors exhibit slow growth, and their clinical presentation depends on anatomical location [3]. Our patient had several vertebrae affected in the thoracic and lumbosacral spine of unknown duration, and his symptoms of back pain started after a fall. We suspect his clinical course was indolent, and the recent fall prompted radiographic examination. CT and MRI of the spine revealed multiple radiolucencies, which were incidental findings.

The lytic bone lesions caused by brown tumors can easily be confused for an underlying neoplastic disease. Indeed, it can imitate multiple myeloma, giant cell granuloma, or metastases [2,10]. However, brown tumors do not exhibit malignant potentiality [10]. Therefore, malignancy was high on our differential diagnosis list, and ruling it in or out was a priority. The final diagnosis is made by tissue examination [4], which commonly reveals increased osteoclastic activity, multinucleated giant cells, fibroblast proliferation, and hemosiderin deposition [3,4]. The loose connective tissue is replaced by cortical and trabecular bone [6].

Medical management of the brown tumor consists of treating the sHPTH with phosphate binders, vitamin D analogs, and calcimimetics, and limiting dietary phosphorus intake [6]. If medical treatment fails, then the patient should be referred for parathyroidectomy [6]. Bisphosphonate use was reported in some cases of spinal implication before the surgical intervention to potentially suppress an increase in the tumor size post-parathyroidectomy. The ultimate treatment is subtotal or total parathyroidectomy, which prompts remineralization of the bone in months to a few years [4].

Without appropriate treatment, the brown tumor can cause bone destruction, fractures [10], and if localized in the spine, cord compression can occur with severe neurological deficits requiring surgical decompression [4,9].

## Conclusions

Diagnosis of brown tumors can be easily overlooked since their appearance can mimic more serious diseases, such as malignancy. A biopsy is a useful procedure that helps distinguish a benign lesion from a neoplasm and can guide the appropriate treatment. Our patient had multilevel spine involvement, which in some instances can lead to spinal cord compression. Timely detection and treatment are essential.
